# Cellular and Molecular Mechanisms of Anti-Phospholipid Syndrome

**DOI:** 10.3389/fimmu.2018.00969

**Published:** 2018-05-07

**Authors:** Marko Radic, Debendra Pattanaik

**Affiliations:** ^1^Department of Microbiology, Immunology and Biochemistry, University of Tennessee Health Science Center, Memphis, TN, United States; ^2^Division of Rheumatology, Department of Medicine, University of Tennessee Health Science Center, Memphis, TN, United States

**Keywords:** anti-phospholipid syndrome, systemic lupus erythematosus, neutrophil extracellular traps, autoantibodies, beta2 glycoprotein I, phospholipids, coagulation protein disorders, thrombosis

## Abstract

The primary anti-phospholipid syndrome (APS) is characterized by the production of antibodies that bind the phospholipid-binding protein β2 glycoprotein I (β2GPI) or that directly recognize negatively charged membrane phospholipids in a manner that may contribute to arterial or venous thrombosis. Clinically, the binding of antibodies to β2GPI could contribute to pathogenesis by formation of immune complexes or modification of coagulation steps that operate along cell surfaces. However, additional events are likely to play a role in pathogenesis, including platelet and endothelial cell activation. Recent studies focus on neutrophil release of chromatin in the form of neutrophil extracellular traps as an important disease contributor. Jointly, the participation of both the innate and adaptive arms of the immune system in aspects of the APS make the complete understanding of crucial steps in pathogenesis extremely difficult. Only coordinated and comprehensive analyses, carried out in different clinical and research settings, are likely to advance the understanding of this complex disease condition.

## Introduction

**Figure 1:**
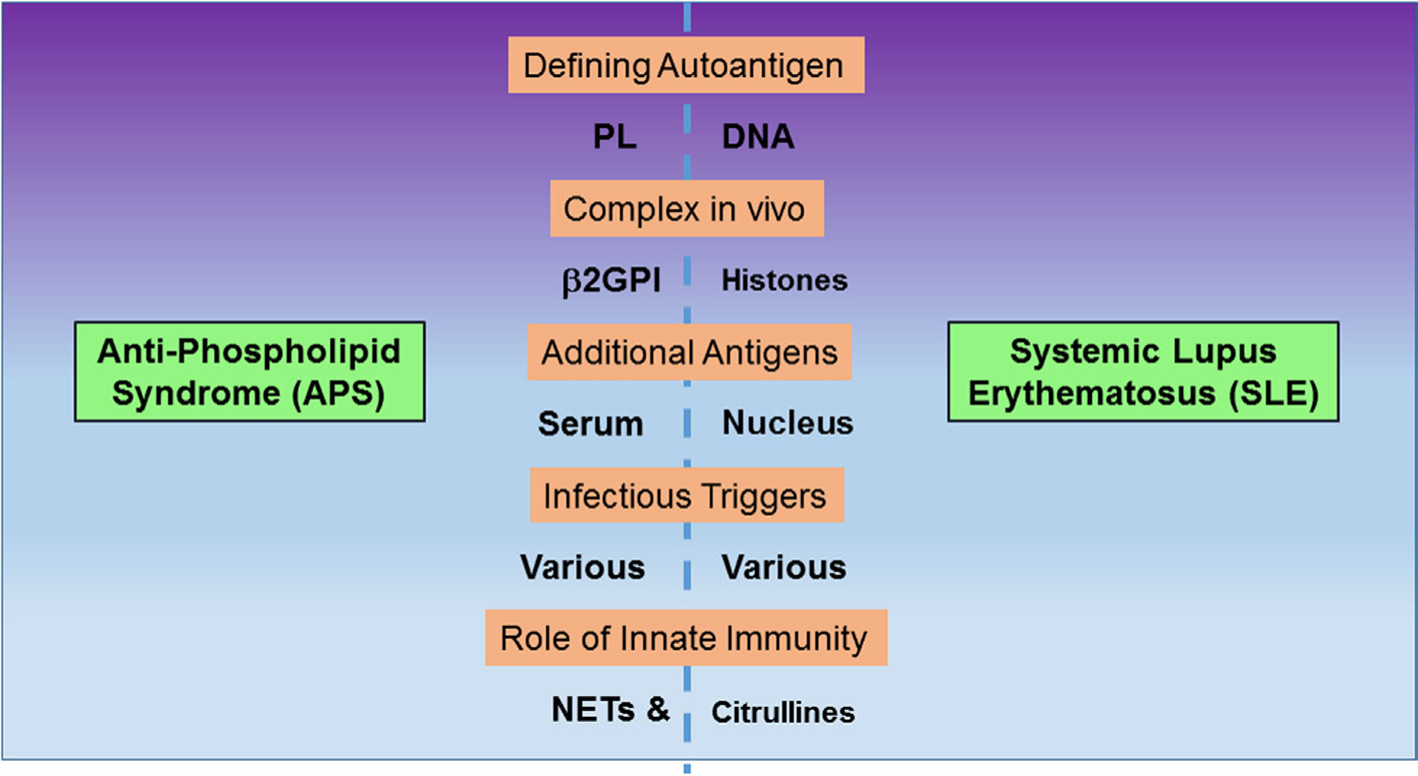
Comparison of features between anti-phospholipid syndrome (APS) and systemic lupus erythematosus (SLE). The two autoimmune disorders exhibit autoantibodies to negatively charged, non-protein antigens, phospholipids (PL), and DNA. However, autoantibodies also recognize complexes between PL and β2GPI or DNA and histones, respectively. Additional autoantibody targets include other serum proteins in APS and nuclear proteins in SLE. Both disorders are potentially triggered by infections, and innate immunity contributes to pathogenesis, as neutrophil extracellular traps (NETs) form integral components of thrombi *in vivo* and citrullinated histones are prominent anti-citrullinated protein autoantibodies.

Anti-phospholipid syndrome (APS) and systemic lupus erythematosus (SLE) are two autoimmune disorders that have puzzled researchers for decades (1–3). The two disorders have a range of shared clinical manifestations and can occur together in the same individual, often after a period of exclusive APS or SLE manifestations. Therefore, it is possible to consider them as different points of departure along a continuum of potential clinical manifestations. According to that view, secondary APS may arise as consequence of a worsening overall disease presentation. Antibodies to phospholipids (PL) and DNA are emblematic of the two disorders. Here, we highlight similarities and differences between the two disorders (Figure 1) in order to argue that discoveries across related research fields will help advance understanding of the unifying factors in their pathogenesis and help explain their notable overlap in presentation. Below, we raise important and as yet unanswered questions that address the relation between external stimuli or insults to the immune system, the diverse and often unique immune responses to these stimuli, the characteristics of the resulting antigen specificities, and the initial break in tolerance mechanisms. Importantly, we summarize how autoantibody binding shapes the observed pathology of the disorders and how it informs the search for new therapies.

A striking feature of APS and SLE is the nature of the defining antigens. Both DNA and PL are among the most abundant and pervasive antigens in the body and both are highly negatively charged. It is not surprising that charge interactions play an important role in DNA/PL recognition and that antibodies with positively charged residues in the complementarity determining regions are positively selected to recognize both autoantigens (4, 5). In fact, the similar charge distribution is, in part, one reason for the observed cross-reactivity between anti-PL and anti-DNA antibodies (6). Both DNA and the negatively charged PL are usually shielded from the humoral immune system by the cell membrane but become externalized during cell death on the surface of apoptotic cells (6, 7). In other forms of cell death, such as necrosis or NETosis, a recently defined neutrophil death (8) that involves the dispersal of chromatin in the form of neutrophil extracellular traps (NETs), DNA and negatively charged PL are also likely to be externalized and to become accessible to antibodies. Therefore, it is reasonable to conclude that cell death contributes antigens that stimulate the anti-self response in APS and SLE (9).

Additional features of both autoantigens include the fact that they exist as multi-molecular complexes *in vivo*. As is the case for most charged macromolecules in the body, both DNA and PL are neutralized by basic proteins that carry countercharges, such as the positively charged histones for DNA and β2 glycoprotein I (β2GPI) for PL. Interestingly, DNA and PL are also recognized by other abundant serum proteins including C-reactive protein, serum amyloid protein, collectins, and pentraxins (9). These proteins contribute to scavenge and clear apoptotic cell debris and possibly the remnants of other forms of cell death. More recently, β2GPI was observed to bind microvesicles and thus potentially participate in the signal transduction mediated by these subcellular particles (10). By several pathways, β2GPI contributes to the physiological clearance of dead cells (11) and it may serve to restore homeostasis following an insult to the body in the form of an infection or other tissue injury.

Depending on the precise molecular interactions, antibody binding to β2GPI could either assist in the clearance of dead cells or derail the normal course of apoptotic cell removal. Obstructive binding of antibodies to β2GPI, therefore, could delay clearance of cell debris and increase the risk of apoptotic cell dispersal. In that way, anti-β2GPI could promote the broader autoantibody reactivity to autoantigens displayed on apoptotic cells, such as DNA and chromatin. The binding of anti-β2GPI to β2GPI may interfere with apoptotic cell recognition and clearance, thus favoring the generation of autoantibody specificities that are indicative of lupus or related autoimmune diseases. Because APS shares certain vascular manifestations not only with Wegener’s granulomatosis and polyarteritis nodosa but also other vasculitis conditions (12), a deeper insight into the autoreactivity in APS may shed light on the mechanisms shared by this broad constellation of autoimmune disorders. Detection of anti-PL prior to diagnosis in subsequent patients with SLE is associated with more severe SLE manifestations, including renal disease, thrombocytopenia, and thrombosis (13). Experimental support for the initiating role of anti-β2GPI antibodies in a broader autoimmune response derives from mice immunized with human β2GPI in lipopolysaccharide (LPS) adjuvant, which exhibit delayed clearance of apoptotic cells and, over time, an increase in autoantibody binding to nuclear autoantigens (14). Importantly, T cell recognition of β2GPI peptides may contribute to epitope spread in mice and humans that may include typical SLE autoantigens (15).

An intriguing open question is whether infections induce anti-DNA and anti-PL antibodies. This may be the case because microbes and the host may share cross-reactive antigens; APS was initially discovered due to a false-positive test for syphilis (16). Alternatively, the infectious process may induce the exposure of self-molecules on the cell surface. In the latter case, posttranslational modifications (PTM) that characterize the innate response to infections may determine the reactivity profile of the induced autoantibodies. Such is indeed the case, as autoantibodies frequently target the specific PTM that arise during an immune response to infections. One notable example is the induction of autoantibodies to self-antigens that contain citrulline residues (17). Citrullines are produced by peptidylarginine deiminases (PADs) that convert certain arginine residues in proteins to citrulline residues (18) and become activated in granulocytes that are exposed to infectious or inflammatory stimuli (19). In fact, citrullinated histones are integral components of NETs. Notably, autoantibodies to citrullinated self-proteins are diagnostic for a range of autoimmune disorders, including SLE (20), and NETs appear to play a key role in the formation of thrombi (21–23). Additional PTM may result from infections and affect the binding of APS antibodies to β2GPI, as circulating levels of oxidized β2GPI correlate with the appearance of anti-β2GPI IgG (24).

An additional mechanism may link β2GPI to the pathogenesis of thrombotic events in APS. This may result from the direct binding of β2GPI to endothelial cells and the activation of inflammatory receptors on these cells (25). The direct binding of β2GPI to endothelial cells, a process that is aided by TLR4, directly activates endothelia. Similarly, Laplante et al. (26) showed in a carotid artery injury model that anti-β2GPI activation of endothelial cells is dependent on TLR4. The binding of β2GPI to TLR4 is enhanced by LPS and may reflect a possible scavenging of LPS. Conversely, anti-β2GPI antibodies enhance the production of pro-thrombotic and pro-inflammatory responses in blood vessels, a mechanism that, in part, is driven by activation of the nuclear factor κB (NF-κB) and AP1 signaling pathways (27). In the following sections, we focus on APS and leave a more detailed comparison to SLE for a separate venue.

## The Fundamentals of APS

Anti-phospholipid syndrome is characterized by vascular thromboembolism, miscarriages, and other pregnancy comorbidities (1). The presence of anti-PL, which include anti-cardiolipin (anti-CL) anti-β2GPI antibodies, and lupus anticoagulant (LA), are the *sine qua non* for the diagnosis of APS (28). Vascular thrombosis, which can affect venous, arterial, or small blood vessels, is identified by histopathologic or imaging analysis. These antibodies are essential for the diagnosis and likely to play a pathogenic role in various disease manifestations (29). Thrombotic events in APS are rarely accompanied by histological evidence of vessel wall inflammation, yet many APS patients have underlying systemic autoimmune disease (30). APS pathogenesis clearly involves inflammatory pathways in endothelial cells, monocytes, and neutrophils and a variety of intercellular interactions promotes disease progression.

Anti-phospholipid syndrome-associated manifestations may include thrombocytopenia, livedo reticularis, skin ulcers, cardiac valve and kidney damage, pulmonary hemorrhage, and certain neurological manifestations (31). Patients experiencing these manifestations generally do not improve with anticoagulation therapy, suggesting that additional pathophysiologic processes may cause these outcomes of thromboembolism.

Initially, anti-PL antibodies were thought to bind directly to PL but later it was found that anti-PL may recognize negatively charged PL indirectly *via* PL-binding plasma proteins (32, 33). Anti-PL antibodies are quite heterogeneous and react with PL, PL-binding proteins, and their complexes (33). β2GPI is the main binding cofactor of these antibodies (34) and detection of anti-β2GPI has the greatest clinical significance (33). The analysis of antibody binding to β2GPI must take into account that β2GPI consists of five independently folded domains, including domain V, which resembles a “hook” and interacts with the PLs in the cell bilayer, and, at the opposite end, domain I, which is recognized by most clinically relevant antibodies in APS (35).

Depending on the redox state of the extracellular milieu, domains I and V expose different epitope surfaces for antibody binding. A tight interaction between domains I and V, which defines the circular form of β2GPI *in vivo*, shields various epitopes on domain I. The dissociation between the two domains gives rise to the linear, fishhook-like structure of β2GPI in which the domain I epitopes are exposed (36). Cysteine residues at positions 288 and 326 of domain V, which either remain as free thiols or form a disulfide bond, control the conversion between the two alternative *in vivo* conformations. In the plasma of healthy individuals, β_2_GPI occurs in the free thiol form, which folds into a ring configuration and blocks antibody access to the principal domain I epitopes (37). Oxidative stress unfolds the ring conformation of β2GPI, exposing the normally shielded antigenic determinants of domain I, which form epitopes for pathogenic antibodies (36, 38). This form inserts with domain V into the cell bilayer of anionic PL. Raimondo et al. determined a strong positive correlation between IgG anti-domain I and the proportion of oxidized β2GPI, but not with IgM or IgA anti-domain I (24). This observation suggests that either anti-domain I IgG stabilizes the extended, oxidized form of β2GPI or that chronic inflammatory conditions lead to an abundance of oxidized β2GPI that stimulates the production of anti-domain I IgG.

Other potential antigen targets include phosphatidylserine, tissue plasminogen activator, plasmin, thrombin, prothrombin, antithrombin III, activated protein C, and annexin V (33). The diversity of potential antigens argues for the existence of “seronegative” APS and some investigators have disputed the primary significance of anti-β2GPI antibodies (39). Indeed, some cofactor independent antibodies can induce thrombus formation in a mouse model (40). Overall, autoantibodies in APS, as the disorder itself, are thought to arise due to a pernicious interaction between environmental factors and increased genetic predisposition to the disease (41).

There is no general agreement on the mechanisms that contribute to thrombotic complications in APS (42). Inconsistencies that prevent a consensus from emerging are: a. the differences between patient populations used to isolate the autoantibodies, b. the specificity of the antibodies used, and c. the experimental model in which the antibodies are tested (43). Anti-PL antibodies increase the risk of thrombosis through different mechanisms that go beyond a simple dysregulation of coagulation pathways (44). It is likely that mechanisms other than simple vascular thrombosis contribute to various APS manifestation. The fact that thrombotic events occur sporadically in spite of persistently high level of anti-PL antibodies suggests that factors in addition to anti-PL antibodies are required for thrombosis to arise (45).

## Genetic Factors Predisposing to APS

A genetic basis for anti-PL antibodies was suspected by Harvey and Shulman from their finding of familial clustering of false-positive tests for syphilis (46). Anti-CL antibodies occur more frequently in first-degree relatives of SLE or primary APS patients than in unrelated control individuals, indicating that a genetic susceptibility favors the expression of anti-PL. Extended kinships with elevated expression of anti-PL were analyzed with regard to APS clinical presentation and provided evidence for a familial form of APS (47, 48). In another study, Goel et al. examined possible modes of genetic inheritance and noted the potential involvement of candidate genes. Their study, which involved 30 family members of APS patients, failed to confirm the contribution of several candidate genes to the disorder (49).

The combination of HLA-DQw7 (HLA-DQB1*0301) with HLA-DR4 or HLA-DR5 was significantly elevated in patients with SLE and LA as compared to 139 race-matched controls (50). Patients also expressed other HLA-DQB1 alleles from which the authors deduced a shared amino acid sequence, TRAELDT, which they proposed to constitute a potential autoantibody epitope (50). In another study, DR4 and DRw53 occurred with increased frequency in patients with primary APS (51), and a study of 577 European SLE patients presenting with anti-CL antibodies found a positive association with DPB1*1501 (*P* value: 0.005, OR 7.4), and DPB1*2301 (*P* value: 0.009, OR 3.3). Anti β_2_GPI antibody was positively associated with DPB1*0301 (*P* value: 0.01, OR 1.9), and DPB1*1901 (*P* value: 0.004, OR 8.1). The authors conclude that the genetic risk of anti-PL antibodies—along with other clinical manifestations of APS—may be increased in SLE patients who are positive for certain HLA-DPB1 alleles (52). In Japanese patients with APS secondary to SLE, DRB1*09 has been linked to anti-CL (53). In Caucasians and Mexican Americans, HLA-DQ8 (DQB1*0302) and related HLA-DR4 haplotypes may predispose to anti-β2GPI, whereas British patients with primary APS show an association between anti-β2GPI and the HLA-DRB1*1302 and DQB1*0604/0605 (50, 54–56). Furthermore, C4A or C4B null alleles may associate with the presence of anti-CL antibodies in African-American populations (57). Notably, a polymorphism in domain V of β2GPI is observed more frequently in APS patients with anti-β2GPI antibodies than in matched controls (58, 59). Genetic polymorphisms have also been linked to thrombosis in APS patients. These polymorphisms range from variants of tissue factor (TF) pathway inhibitor, type-I plasminogen activator inhibitor, tumor necrosis factor α (TNFα), annexin A5, p-selectin, p-selectin glycoprotein ligand-1 (PSGL-1), platelet Fc receptor IIa, platelet glycoproteins GP Ia/IIa and GP IIb/IIIa, thrombomodulin, factor XIII, methylenetetrahydrofolate reductase, toll-like receptor 4, and CD40 (33). In view of the many diverse genetic factors that predispose to APS, a picture of a delicate balance of steps in the coagulation pathway emerges, in which a disequilibrium at any one point may tilt the equation toward thrombosis.

## Genetic Analysis in Model Systems

The first evidence that genetics contributes to pathogenic anti-PL in APS came from studies in mice. The spontaneous production of pathogenic IgG anti-CL that depend on β2GP for binding to CL occurs in NZW x BXSB F1 (W/B F1) male mice (60). W/B F1 mice develop autoantibodies to negatively charged PLs, including phosphatidylserine and phosphatidylinositol, and generate circulating immune complexes, which ultimately result in glomerulonephritis. The pathogenic anti-CL antibodies preferentially use certain V_H_ and V_κ_ genes, whereas non-pathogenic anti-CL antibodies use more heterogeneous V genes (61). Microsatellite markers have enabled genetic analysis of BXSB alleles that affect production of anti-CL and anti-platelet antibodies, cytopenia, and coronary artery disease in W/B F1 male offspring (62). Disease was dependent on two dominant alleles that acted as complementary genes and localized to separate chromosomes. Anti-platelet antibodies and thrombocytopenia were genetically and mechanistically linked but anti-CL and myocardial infarction depended on independent genetic contributions, suggesting that genetics of APS is complex (62). In another mouse model, the MRL-lpr/lpr mice, the specificity of a monoclonal anti-CL was shown to depend on stochastic events, including somatic mutations in the V_H_ gene, indicating that failure in peripheral tolerance mechanisms followed by antigen-driven selection and clonal expansion contribute to this autoreactivity (61).

Papalardo et al. demonstrated that pathogenic anti-PL and clinical manifestations of APS depend, in part, on particular MHC-II alleles (63). Wild-type mice, or mice that expressed human DR4, DQ6, or DQ8 genes, but not MHC-II knockout mice, produced thrombogenic anti-PL and TF after immunization with human β2GPI. In addition, in wild-type C57BL/6J mice, anti-CL antibodies were not β2GPI dependent and instead showed diminished binding to CL in the presence of the β2GPI cofactor (64). This study suggested the importance of certain MHC class II haplotypes in determining the levels of anti-PL antibodies and their pathogenic capacity.

## Infections as APS Triggers

Infections are potential inducing factors for the production of autoantibodies in APS (65). Various infectious agents have been linked to the pathogenesis of APS but a definitive proof is still lacking. BALB/c mice infected with *Haemophilus influenzae, Neisseria gonorrhoeae* or immunized with tetanus toxoid developed antibodies to the TLRVYK peptide and anti-β2GPI reactivity (66). Moreover, naïve mice developed features of classic APS after infusion of these antibodies. The hexapeptide TLRVYK is a component of proteins expressed by these microbes and is also recognized by a pathogenic monoclonal anti-β2GPI antibody, suggesting the role of molecular mimicry as the potential cause of development of APS. A literature review revealed that, in people, the development of APS may be linked with HIV, HTLV, HBV, HCV, parvovirus B19, and varicella zoster virus infections (67). Infectious agents may induce autoantibodies through various mechanisms. Possible mechanisms include molecular mimicry, increased secretion of cytokines and chemokines, selective activation or depletion of lymphocyte populations, and exposure of cryptic epitopes due to the induction of cell death (68, 69).

Certain infectious agents may also directly affect the immunogenicity of β2GPI. Patients with APS exhibit a significant increase in oxidized β2GPI (70). Infectious agents could generate conditions that favor reactive oxygen and nitrogen species that may enhance β2GPI oxidation and autoantibody production (71). Medications, such as chlorpromazine, amoxicillin, quinine, chlorothiazide, and propranolol, in addition to oral contraceptives, alpha-interferon and infliximab, may promote the expression of anti-PL antibodies (72). The preferred interpretation of these results is that medications may bind to self-antigens and create new binding determinants, so-called neo-antigens, which may induce autoantibody production (73).

## Endothelial and Platelet Contributions

Cell activation is a key element in the increased thrombotic response (42). Some authors suggested endothelial cells are critical in APS-associated thrombosis (74), whereas others proposed a paradigm shift, which favored a central role of platelets (75). It is also possible that endothelial cells, directly or indirectly, promote the release of pro-thrombotic microparticles (76). This promises to be an exciting area of research in the near future.

## Innate Immunity and NETs

The cellular immune response to infections may be directly responsible for generating conditions that are favorable for the initiation of APS. Although lymphocytes, monocytes, and platelets receive much deserved attention for their role in the pathogenesis of APS, neutrophils contribute in a unique and relevant manner to the development of APS (77). Neutrophils are by far the most abundant leukocyte in the blood and they rapidly respond to inflammatory stimuli (78). Circulating neutrophils attach to activated endothelia, which express adhesion molecules, and invade tissues that harbor infectious organisms or exhibit other signs of inflammation. The neutrophils have alternative mechanisms to combat microbes, including phagocytosis and granule discharge (79). An intriguing antibacterial mechanism is the release of NETs. NETs consist of nuclear chromatin that escapes from the confines of the nucleus and disperses as an amorphous lattice from the cell. The NET fibers attach to various components of neutrophil granules that help to enhance the bactericidal properties of the lattice (80).

Neutrophil extracellular traps are important in the context of APS because APS patient neutrophils are prone to spontaneous NET release (22), and thrombi incorporate NET-derived materials (21–23, 81). *In vitro*, neutrophils respond to incubation with anti-β2GPI antibodies by an intensified NET release (22). In animal models, inhibitors of NET release show promise in reducing thrombus formation, and mice deficient for PAD4, the enzyme that deiminates histones and promotes DNA unraveling in NETs, are resistant to pro-thrombotic stimuli (82). A recent study identified PSGL-1, a neutrophil protein that mediates adhesion to endothelia, as an important regulator of the pro-thrombotic functions of neutrophils, and small molecules that target this protein may hold the key to new therapies for APS (83). Clearly, neutrophil biology in the context of APS warrants further attention and is likely to reveal new and exciting implications for APS pathogenesis.

## Mechanisms of Antibody-Mediated Thrombosis

The pathogenic mechanisms that contribute to thrombus formation have been examined using both *in vitro* and *in vivo* models of APS. Anti-PL antibodies increase thrombus formation in the venous and arterial circulation (84–86). Infusion of autoantibodies from APS patients to mice with injured blood vessels potentiates thrombus formation in a way that suggests a pathogenic role for APS antibodies. Anti-β2GPI IgG autoantibodies, but not IgG depleted of anti-β2GPI reactivity, or normal human IgG, increase thrombus size in a dose-dependent manner (87). Administration of human anti-PL IgG along with LPS causes micro thrombosis in rat model (88). In contrast, infusion of anti-PL antibodies alone into the experimental animal models does not result in spontaneous thrombotic complications, thus suggesting the requirement for priming with a small vascular injury or injection of a low dose of LPS. This is in line with the “Two Hit Hypothesis” (89) that was proposed to account for the clinical observation that, despite the continued presence of anti-PL, thrombotic events are rare. According to the two-hit hypothesis, the anti-PL antibody induces a thrombophilic state, but requires a second condition (e.g., an infection) for clotting to take place. Infusion of purified anti-PL antibodies with or without dimeric β2GPI alters endothelial adhesion molecule expression and leads to a perturbation of vascular function associated with TLR 2 and TLR4 signaling and the upregulation of nitric oxide and TF expression (86, 90–93). As microbes and microbial products signal through TLRs, so it is possible that an infection and anti-PL signaling through the TLRs can additively increase the risk of thrombosis. Thus, infections or inflammation may increase the expression of the anti-PL target or enhance the exposure of previously hidden epitopes (37). None the less, the “two hit hypothesis” does not conform well with the obstetric manifestations of APS, where the anti-PL is the single factor that leads to the increased risk of venous thromboembolism during pregnancy (94), although pregnancy itself may be viewed as the “second hit.”

A recent systematic review and meta-analysis found that LA and anti-CL antibodies are associated with an increased risk of venous thromboembolism [OR = 6.14 (CI 2.74; 13.8) and OR = 1.46 (CI 1.06; 2.03), respectively] (95). All three antibodies show a significant association: ORs for LA, anti-CL, and anti-β2GPI were 3.58 (CI 1.29–9.92), 2.65 (CI 1.75–4.00), and 3.12 (CI 1.51–6.44), respectively, with arterial thrombosis (95). Anti-β2GPI antibodies with LA activity are considered the main culprits for the thromboembolic complications in APS (96). A subgroup of anti-β2GPI antibodies that bind the epitope comprising Gly40-Arg43 (G40-R43) in domain I were shown to act as LA and correlate strongly with thrombosis (34, 97).

Subjects positive for LA, high titers of anti-CL, and anti-β2GPI antibodies (called “triple positives”), more than any other anti-PL profile, have high risks for thrombosis and pregnancy morbidity (98). The risk of recurrent thrombosis in triple-positive patients was around 30% over a 6-year follow-up period. Triple-positive anti-PL patients usually have high titers of antibodies to the major β2GPI epitope on domain I (99). Thus, anti-domain I β2GPI autoantibodies, which frequently present in triple anti-PL-positive patients, confer LA activity, associate with the highest risk of thrombosis (100), predispose to both thrombosis and pregnancy loss (100), and promote thrombosis in mouse models (101). Clearly, a detailed profile of anti-β2GPI antibody specificity and avidity may be useful as a risk stratification resource in the clinic (30).

## Pregnancy Loss

Intraplacental thrombosis leading to poor vascular supply to placenta was thought to be the major pathogenic mechanism but is certainly not the universal mechanism of fetal loss in APS. Other anti-PL antibody-induced pro-inflammatory, complement-mediated pathways, and defective placentation might be playing a role (94). Passive transfer of anti-PL antibodies causes fetal loss due to placental thrombosis and also inhibits trophoblast and decidual cell function *in vitro* and in animal models (102). Anti-PL antibodies, in particular anti-β2GPI antibodies, may compete with the anticoagulant annexin A5 for binding to trophoblast and endothelial cells, thus increasing the risk of placental thrombosis (103). However, the *in vitro* studies may be challenged by the fact that microscopic analysis of tissues from miscarried fetuses or placentas of women with APS rarely show thrombosis (104). This could be related to the timing of the examination of the placental samples, as many of the events may occur early in the pregnancy, and later only residual damage may remain (94).

Complement products, TNFα and CC chemokines, along with other pro-inflammatory mediators, contribute to anti-PL-induced fetal loss in animal models (105). Injection of human anti-PL IgG into naïve mice following embryo implantation caused placental inflammatory changes. Human IgG and mouse complement deposited along the decidua, and a transient increase in blood TNFα coincided with neutrophil infiltration into the tissues (106–108). Studies of animal and human placenta indicate that complement activation by anti-PL may be major contributor to the recurrent pregnancy loss in APS (107). The complement system contributes to fetal loss in the mouse model as either complement inhibition or deficiency of complement components protects the mouse from fetal loss (109).

Complement activation by anti-PL antibodies, which bind decidua and placenta preferentially, may involve the classical and, perhaps, lectin pathways. In the process, potent anaphylatoxins (C3a and C5a) may be generated, leading to the recruitment of inflammatory cells. Further activation of the alternative pathway creates a localized pro-inflammatory amplification loop, which enhances C3a activation and deposition and generates additional anaphylatoxins, thus attracting additional inflammatory cells to the placenta (110). Inflammatory tissue injury is probably mediated by TNF-α, which increases in murine decidua after exposure to anti-PL (108). Additionally, the therapeutic effect of heparin can be traced to inhibition of complement rather than inhibition of coagulation (111). Treatment with unfractionated or low molecular weight heparins protects against pregnancy loss induced by anti-PL antibodies, whereas use of plain anticoagulants, such as hirudin or fondaparinux that have no anti-complement effects, do not protect from pregnancy loss (110).

Nonetheless, investigations have not gathered conclusive evidence to support the pathogenic roles of inflammation and complement deposition in obstetric complications (112). There was no evidence of inflammation in placenta in a mouse model of anti-PL antibody-induced fetal loss following IV administration of human anti-PL IgG before implantation (113). Data from *in vivo* animal models may be inconclusive because of the fact that observations cannot be continuous during the pregnancy and depend on the time chosen for the infusion of the putative pathogenic autoantibodies (94).

Additional mechanisms may be involved in anti-PL-induced fetal loss. Binding of β2GPI-dependent antibodies to human trophoblasts inhibits cell proliferation and syncitia formation, decreases production of chorionic gonadotrophin, perturbs secretion of growth factors, and induces apoptosis (114). Moreover, β2GPI-dependent antibodies may impair the expression of cell adhesion molecules, such as integrins and cadherins, in trophoblastic and decidual cells that perturb function at the maternal side of the placenta (115). Defects in endometrial differentiation, including the impaired expression of complement decay-accelerating factor (also known as CD55), arise and are evident on endometrial biopsies. Such alterations may compromise implantation, if they occur at or before conception. After conception, endometrial defects are likely to predispose to complement-mediated pregnancy failure (116).

Anti-PL greatly increase the risk of preeclampsia. A recent study concluded that anti-PL act, in part, by compromising the mitochondria in the syncytiotrophoblast and increasing the amount of mitochondrial DNA released *via* placental vesicles (117). The vesicles may increase the risk of preeclampsia because the mitochondrial DNA, which is recognized as a DAMP by TLR-9, may activate endothelial cells. If this concept is confirmed, then pharmaceutical intervention aimed at reducing placental vesicles and the signaling by mitochondrial DNA through TLR-9 may have the potential to lessen the adverse consequences of anti-PL in pregnancy (117).

## Immune Signaling Pathways

It is not clear how binding of anti-PL antibodies to endothelial cells may lead to cell activation, as no clear cellular activation pathway has been identified. Candidate interactions include the binding of the anti-PL-β2GPI complex to TLR 2 or 4, the binding of annexin A2, or mediation of the low density lipoprotein receptor-related protein 8, followed by activation of a signal transduction pathway inside the cells. In each case, a more pro-thrombotic cell phenotype may be the outcome (42). Activation of individual or sets of receptors are possible (118). A recent study has shown that antibody uptake is essential for anti-PL antibody-induced cellular signaling (119). MyD88 and TRAF6-dependent signaling, as well as NF-κB and p38 mitogen-activated protein kinase signaling, may be involved downstream from anti-PL binding to β2GPI on the cell surface (114). However, it is not clear whether clinical manifestations differ depending on which cell signaling pathways are engaged, or whether different anti-PL subpopulations have different effects on cell activation (94).

Activation of the mechanistic target of rapamycin (mTOR) pathway plays a role in endothelial proliferation and intimal hyperplasia in anti-PL-positive patients, which leads to multiple potential outcomes, including micro thrombosis, peripheral ischemia, skin ulcers, diffuse alveolar hemorrhage, or anti-PL nephropathy. IgG antibodies from APS patients, when incubated with vascular endothelial cells, stimulate the mammalian/mTOR through the phosphatidylinositol 3-kinase–AKT pathway (120) leading to cell proliferation. The authors showed that sirolimus, a mTOR complex inhibitor reduced endothelial cell proliferation and vascular lesions among patients with APS nephropathy, who required transplantation, as compared with patients with anti-PL antibodies, who did not receive sirolimus. Furthermore, *in vitro* studies have shown that treatment of anti-β_2_GPI/β_2_GPI or APS-IgG/β_2_GPI complex could markedly induce mTOR activation as well as expression of TF and IL-8 in THP-1 cells (a human monocytic cell line) or primary monocytes. The mTOR inhibitor rapamycin (100 nM) could attenuate the elevated expression of TF and IL-8 (121).

## Medications and Potential Therapies

A necessary step in anti-PL-mediated thrombosis and fetal loss seems to be the activation of complement, as discussed above. The activation of the classical complement pathway in APS-associated thrombosis is evident from mouse studies (88, 122). Activation of complement by anti-PL autoantibodies generates C5a, which attracts and activates neutrophils and enhances expression of TF (123). Conversely, mice treated with APS patient IgG had higher titers of anti-CL antibodies and anti-β2GPI leading to thrombosis; subsequently, they developed larger thrombi and higher soluble TF activity than controls. The recombinant C5 activation inhibitor rEV576 (coversin) reduced thrombus formation and suppressed TF activity from cells treated with IgG-APS (124, 125). The murine studies are in agreement with human studies. In a study of 186 patients, levels of fragments Bb and C3a were significantly increased compared to normal controls (126). APS patients who suffered from venous thromboembolism had significantly increased complement activation compared to normal controls, which Rivaroxaban effectively reduced (127). Mildly reduced complement levels (C3, C5), perhaps indicating complement consumption, occur in some APS patients (128), although this may not be a consistent feature of the syndrome (94). Supporting the role of complement, case studies indicate the benefits of C5-inhibitor eculizumab in preventing APS-associated thrombotic microangiopathy, a complication of renal transplantation, as well as for treatment of patients with acute catastrophic APS (129, 130).

**Figure 2:**
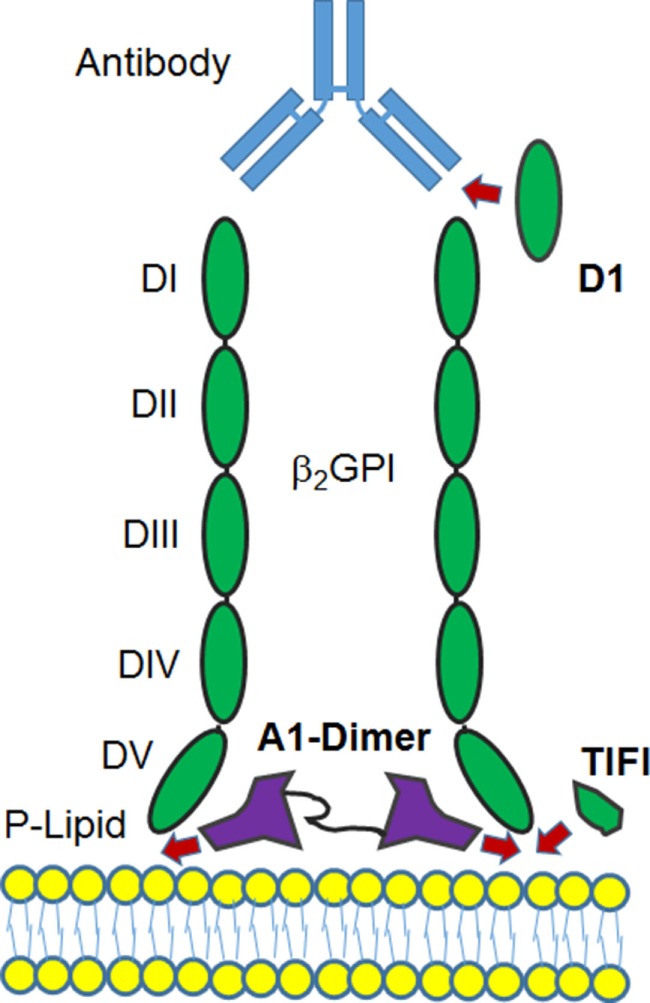
Diagram of potential sites where binding of β2GPI to a phospholipid membrane may be disrupted. Recombinant versions of the D1, a portion of the DV domain (TIFI) or a dimer of the ApoER2 domain A1 are shown to indicate where the formation of the β2GPI-anti-β2GPI complex at the cell surface may be inhibited for therapeutic benefit. For details, see main text.

Additional approaches have involved synthetic peptides (Figure 2). TIFI is a 20 amino acid synthetic peptide that shares similarity with the β2GPI PL-binding site. Administration of the peptide prevents anti-PL-mediated thrombosis *in vivo*, and, as expected, TIFI inhibits the binding of β2GPI to human endothelial cells *in vitro* (131). Infusions of TIFI protected pregnant mice from human anti-PL-induced fetal loss (132), thus providing evidence for the detrimental effect of β2GPI–anti-β2GPI complexes binding to trophoblasts in anti-PL-induced fetal loss (133). Similarly, the recombinant DI domain of β2GPI, the major anti-PL antibody target in APS, could inhibit experimental thrombus development in mice infused with APS patient IgG (134). The observation that β2GPI binds avidly to the ApoER2 A1 domain, the main LDL binding domain 1 (92), was the impetus to construct and test the recombinant dimer of A1 as an effective inhibitor of the pro-thrombotic functions of anti-β2GPI antibodies in mice (135). The successful deployment of each of these three recombinant protein domains (and their variants) raises the possibility that biologic therapies based on these peptide structures (Figure 2) may be developed in the near future.

Because neutrophils likely exert a unique and important function in APS pathogenesis, a range of approaches that limit neutrophil activation and NET release may move into the spotlight as targeted treatments for patients with APS. For example, *N*-acetyl cysteine, an effective scavenger of ROS that reduces the release of NETs *in vitro* and inhibits mTOR in T cells, has shown promise in SLE trials (136). Similarly, inhibitors of myeloperoxidase, a granule component in neutrophils that may catalyze reactions leading to NET release, have been used in patients with vasculitis and may be considered candidates for trials in APS (137). Moreover, the specific TLR4 inhibitor, TAK-242, which acts upstream of mTOR to reduce NET release and inhibit ROS production in neutrophils, has shown potential as treatment for APS (121).

In sum, we propose that APS therapy is at the doorstep of its most exciting stage. Numerous pathogenic mechanisms have been proposed and experimentally supported, and diagnostic and prognostic measures of APS activity have improved to the point that a broad range of potential therapies have appeared on the horizon and could soon advance through regulatory tests toward a safe and effective use in the clinics.

## Author Contributions

MR provided initial planning and wrote sections of the manuscript, edited the text, and gave final approval. DP participated in the planning and writing of sections of the manuscript, edited the text, and gave final approval.

## Conflict of Interest Statement

The authors declare that the research was conducted in the absence of any commercial or financial relationships that could be construed as a potential conflict of interest.
